# Supplementary material for: Noninvasive brain stimulation combined with evidence-based psychotherapy for psychiatric disorders: A meta-analysis of optimal implementation parameters
Source: Neurosci Biobehav Rev. Author manuscript; Available in PMC 2026 Sep 8. (PMC13551994; doi:10.1016/j.neubiorev.2026.106786)
Supplement: Supplementary file 1 [file NIHMS2207750-supplement-Supplementary_Material.pdf]

SUPPLEMENTARY MATERIAL

This supplementary material provides additional detail on the meta-analysis of noninvasive brain stimulation (NIBS) combined with evidence-based psychotherapy for psychiatric disorders. The contents include:

- **Supplementary Figures S1–S5:** Forest plots stratified by key moderator variables, including diagnostic category, psychotherapy type, level of evidence supporting psychotherapy, administration method, and interactions between NIBS modality and psychotherapy category.
- **Supplementary Table S1:** Detailed study population characteristics and outcome measures for all 28 included randomized controlled trials (31 independent comparison groups).
- **Supplementary Table S2:** NIBS parameters including modality (rTMS or tDCS), timing relative to psychotherapy, anatomical target, targeting method, stimulation intensity, protocol specifications, and number of sessions.
- **Supplementary Table S3:** Psychotherapy parameters including therapy type (CBT, exposure, mindfulness), delivery method (human, computerized, or hybrid), concurrent medication allowance, adherence monitoring, number of sessions, and session duration.

All analyses used random-effects models to account for between-study heterogeneity. Standardized mean differences (SMD) are reported with 95% confidence intervals, with negative values indicating superiority of active NIBS combined with psychotherapy over sham NIBS combined with psychotherapy.

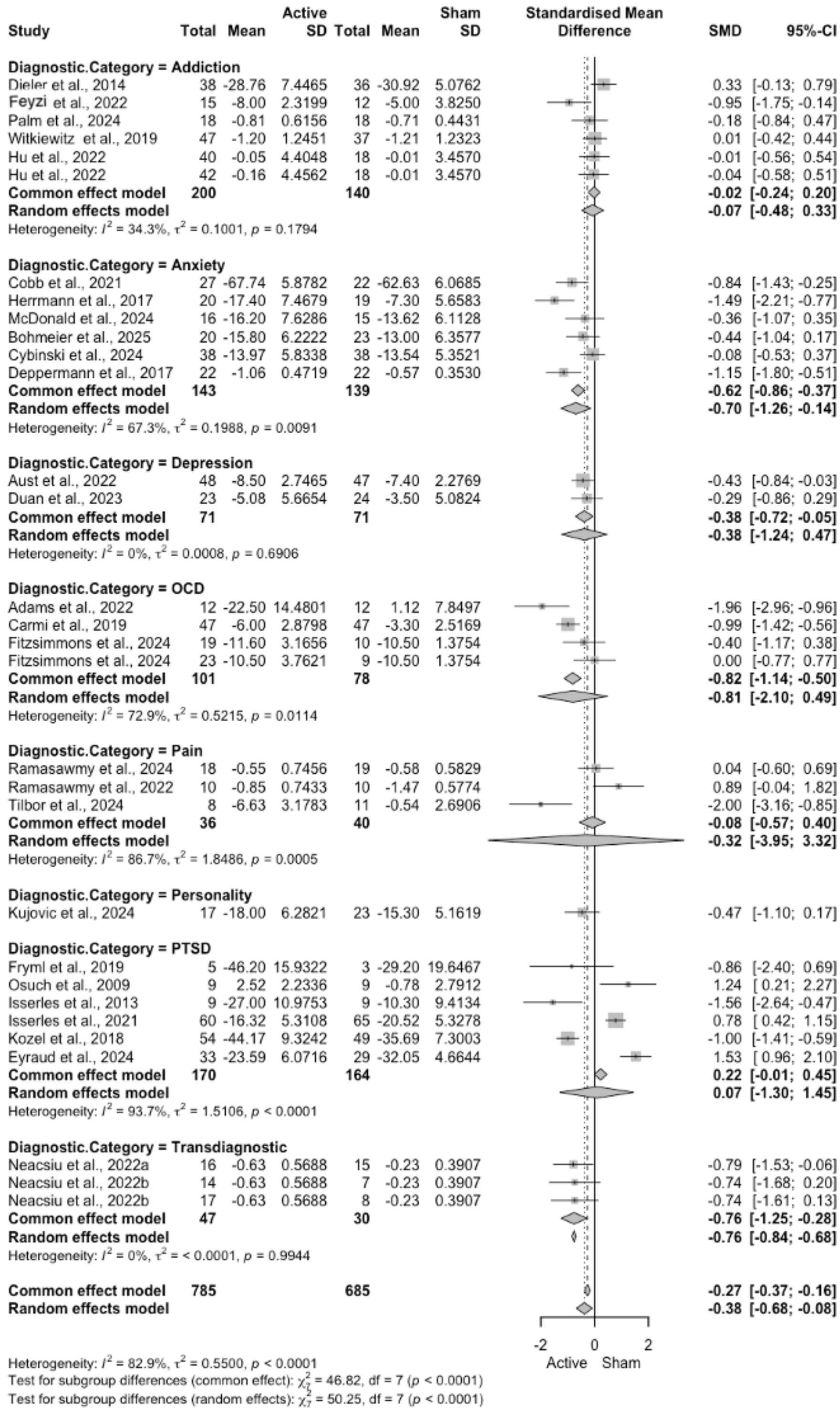

Figure S1: **Primary outcome forest plot stratified by diagnostic category.** Standardized mean differences (SMD; negative values favor active NIBS + psychotherapy) are shown for each trial with 95% confidence intervals, pooled within diagnostic categories using random-effects models. Significant effects were observed only for anxiety disorders ($\mathrm{SMD} = -0.70$, 95% CI: [-1.26; -0.14], $p < 0.05$). No significant effects were found for addiction, depression, OCD, pain, personality disorders, PTSD, or transdiagnostic samples. Heterogeneity (I^2) and p-values are reported for each diagnostic subgroup. The overall pooled effect across all diagnoses ($\mathrm{SMD} = -0.38$, 95% CI: [-0.68; -0.08]) is shown at the bottom.

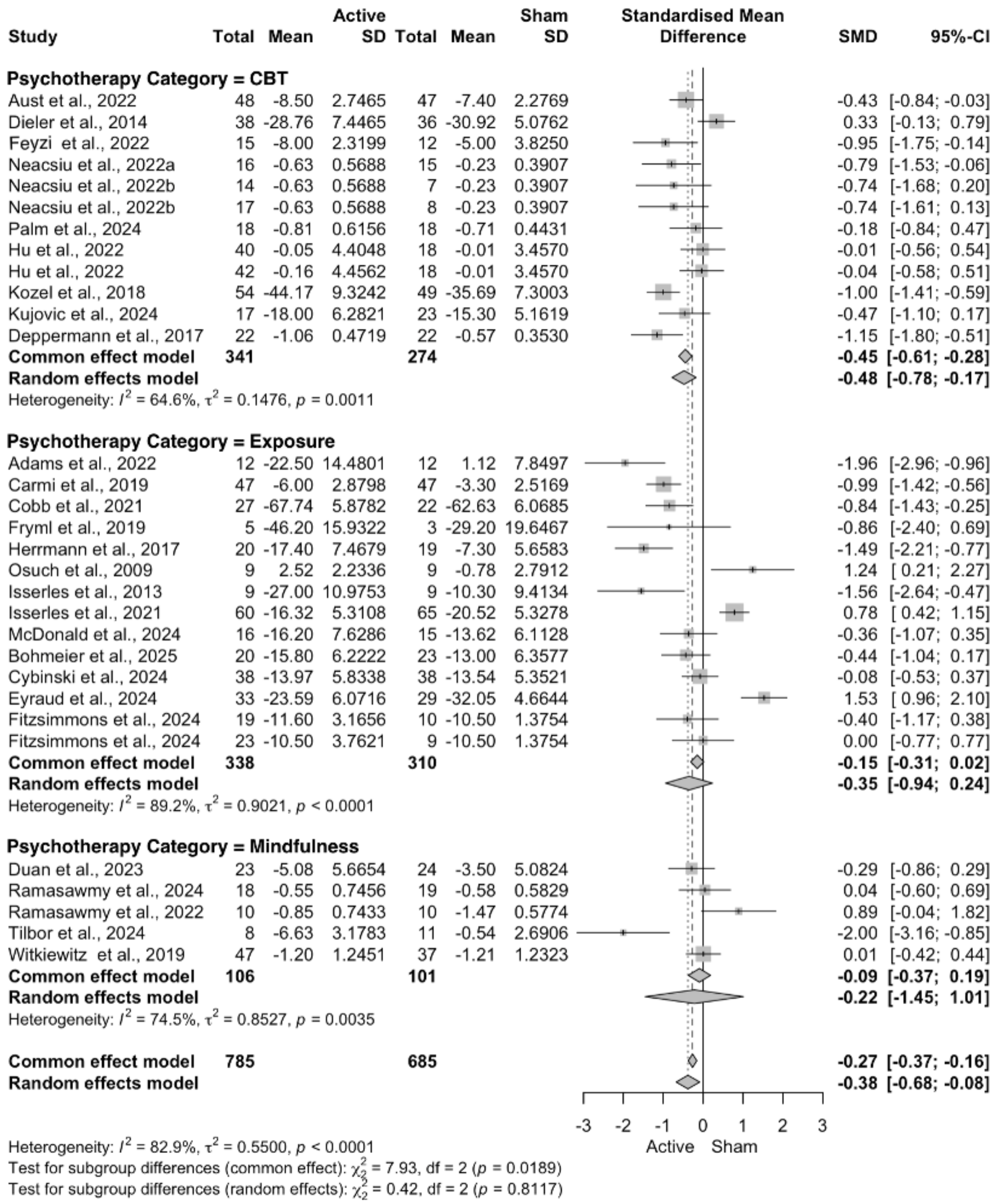

Figure S2: **Primary outcome forest plot stratified by psychotherapy category.** SMDs pooled within psychotherapy categories. Cognitive behavioral therapy (CBT) showed significant improvement (SMD $==-0.48$, 95% CI: $[-0.78; -0.17]$), while exposure therapy (SMD $=-0.35$, 95% CI: $[-0.94; 0.24]$) and mindfulness (SMD $=-0.22$, 95% CI: $[-1.45; 1.01]$) did not.

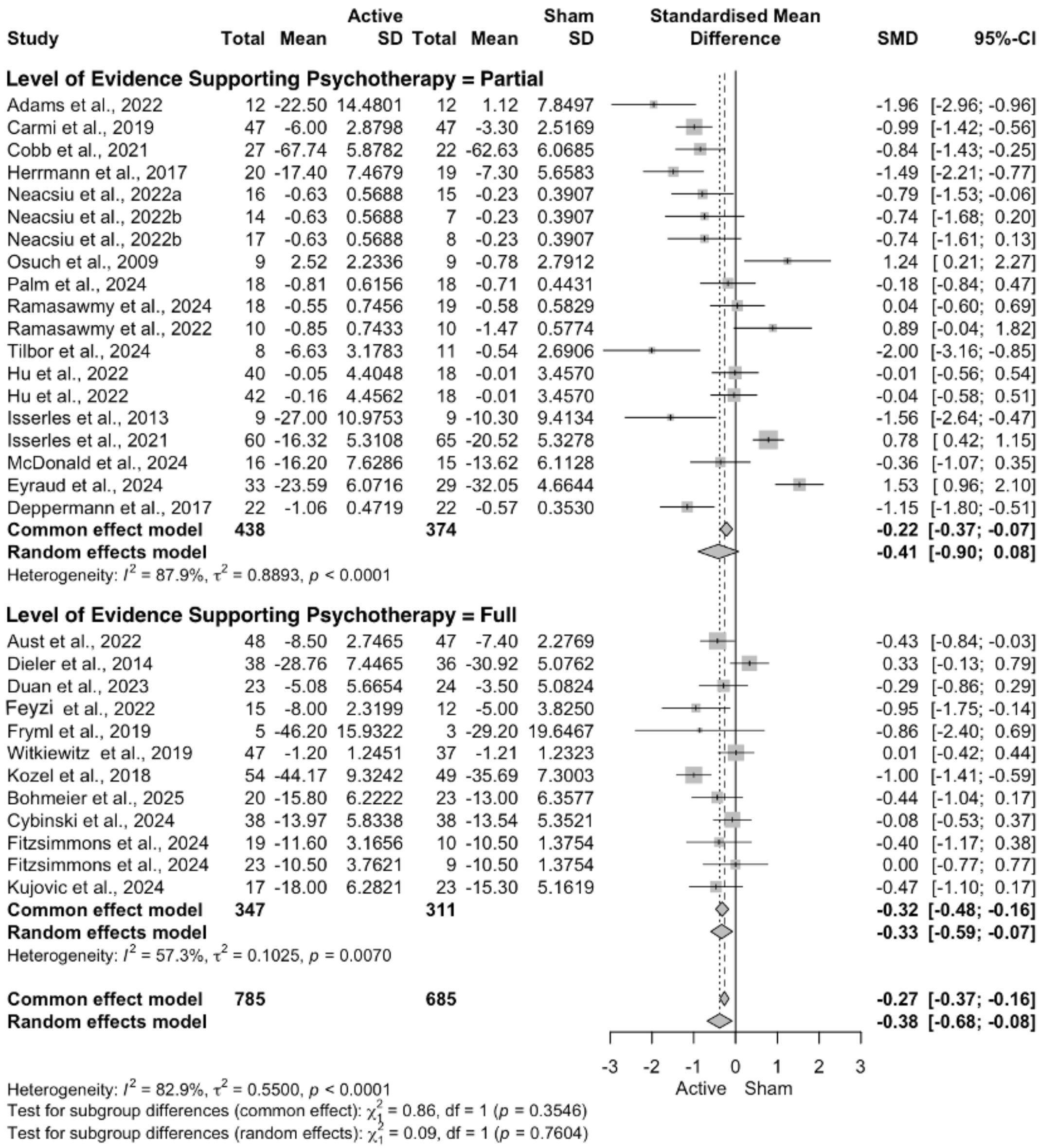

Figure S3: **Primary outcome forest plot stratified by level of evidence supporting the psychotherapy.** SMDs are pooled by whether studies used fully evidence-based psychotherapy (manualized protocols with empirical support) or only partial elements of evidence-based approaches. Although both fully evidence-based ($\mathrm{SMD} = -0.33$, 95% CI: $[-0.59;\ -0.07]$) and partial evidence-based ($\mathrm{SMD} = -0.41$, 95% CI: $[-0.90;\ 0.08]$) psychotherapy showed trends toward improvement, only the fully evidence-based group reached statistical significance.

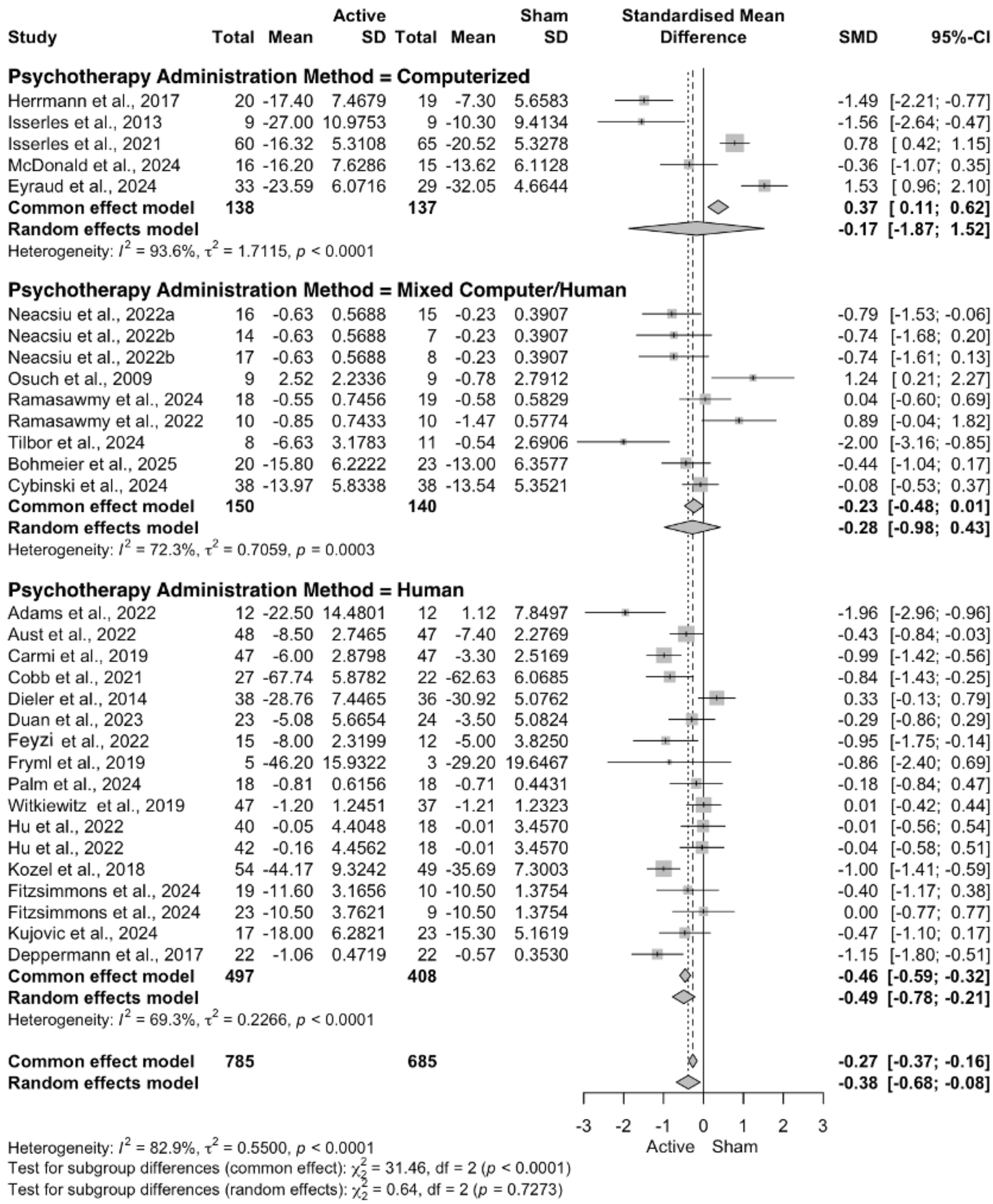

Figure S4: **Primary outcome forest plot stratified by psychotherapy administration method.** SMDs pooled by delivery format. Only human-administered psychotherapy showed significant improvement (SMD $= -0.49$, 95% CI: $[-0.78; -0.21]$), while computerized delivery (SMD $= -0.17$, 95% CI: $[-1.87; 1.52]$) and mixed computer/human formats (SMD $= -0.28$, 95% CI: $[-0.98; 0.43]$) did not reach statistical significance.

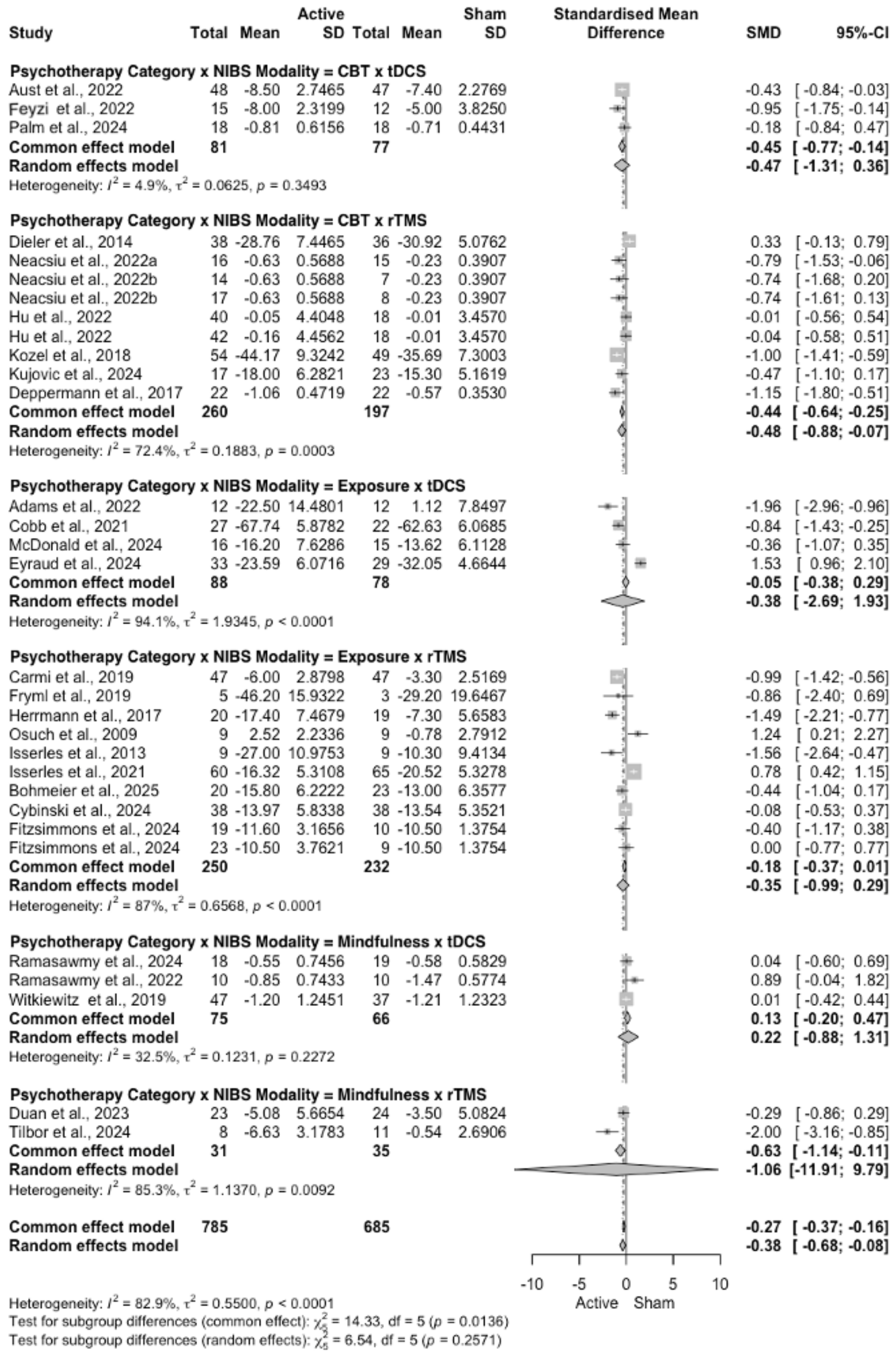

Figure S5: **Primary outcome forest plot for the interaction between NIBS modality and psychotherapy category.** SMDs pooled by the combination of NIBS modality (rTMS or tDCS) and psychotherapy type (CBT, exposure, or mindfulness). Among the combinations with sufficient data for analysis, only CBT paired with rTMS showed significant improvement ($\mathrm{SMD} = -0.48$, 95% CI: $[-0.88; -0.07]$). No significant effects were observed for CBT + tDCS ($\mathrm{SMD} = -0.47$, 95% CI: $[-1.31; 0.36]$), exposure + tDCS ($\mathrm{SMD} = -0.38$, 95% CI: $[-2.69; 1.93]$), exposure + rTMS ($\mathrm{SMD} = -0.35$, 95% CI: $[-0.99; 0.29]$), mindfulness + tDCS ($\mathrm{SMD} = 0.22$, 95% CI: $[-0.88; 1.31]$), or mindfulness + rTMS ($\mathrm{SMD} = -1.06$, 95% CI: $[-11.91; 9.79]$).

Table S1: Study population and outcomes

**Study**	***N*** **(active / sham)**	**Mean age (y) (active / sham)**	**Diagnosis**	**Primary outcome**	**Secondary: Depression**	**Secondary: Anxiety**	**Secondary: QoL**	**Secondary: Executive function**
Adams et al. 2022 [1]	12 / 12	32.4 / 34.4	OCD	SUDS				
Aust et al. 2022 [2]	43 / 42	42.7 / 39.0	MDD	MADRS			SF-36-MH	
Bohmeier et al. 2025 [3]	20 / 23	40.9 / 49.9	Anxiety	AQ (ACRO subscale)	ADS-K	AQ (avoidance)		
Carmi et al. 2019 [4]	47 / 47	41.1 / 36.5	OCD	YBOCS	HAM-D		SDS	
Cobb et al. 2021 [5]	27 / 22	21.6 / 20.7	Anxiety	BAT 1				
Cybinski et al. 2024 [6]	38 / 38	37.8 / 41.6	Anxiety	AQ (ACRO subscale)	ADS-K	ASI-3		
Deppermann et al. 2017 [7]	22 / 22	37.6 / 36.3	Anxiety	PAS–Observer Rated				
Dieler et al. 2014 [8]	38 / 36	46.7 / 46.3	Addiction	SER (Temptation)				
Duan et al. 2023 [9]	23 / 24	58.3 / 53.6	MDD	HAM-D			MBI	MMSE
Eyraud et al. 2024 [10]	30 / 27	31.5 / 35.6	PTSD	CAPS-5	BDI	STAI-A	WHOQoL-BREF	Stroop, N-back
Fayaz Feyzi et al. 2022 [11]	15 / 12	34.0 / 37.0	Addiction	OCDUS				WCST
Fitzsimmons et al. [12] Group 1	23 / 19	31.5 / 40.3	OCD	YBOCS	BDI			
Fitzsimmons et al. [12] Group 2	19 / 19	40.3 / 40.3	OCD	YBOCS	BDI			
Fryml et al. 2019 [13]	5 / 3	27.0 / 30.0	PTSD	CAPS	HAM-D	HAM-A		
Herrmann et al. 2017 [14]	20 / 19	43.2 / 46.6	Anxiety	AQ (anxiety)	ADS			
Hu et al. 2022 [15] Group 1	42 / 37	44.0 / 46.0	Addiction	OCDS	PHQ-9	GAD-7	PSQI	MoCA
Hu et al. 2022 [15] Group 2	40 / 37	48.0 / 46.0	Addiction	OCDS	PHQ-9	GAD-7	PSQI	MoCA
Isserles et al. 2013 [16]	9 / 9	49.0 / 40.4	PTSD	CAPS	HAM-D			
Isserles et al. 2021 [17]	40 / 51	44.8 / 43.7	PTSD	CAPS-5				
Kozel et al. 2018 [18]	32 / 30	34.1 / 32.9	PTSD	CAPS	QIDS			IPF
Kujovic et al. 2024 [19]	17 / 23	24.8 / 25.5	BPD	BSL-23	BDI			GAF
McDonald et al. 2024 [20]	16 / 15	19.8 / 20.0	Anxiety	PRPSA				
Neacsiu et al. 2022a [21]	16 / 15	32.9 / 34.7	Transdiagnostic	DERS		ERQ	OQ-45	WSAS
Neacsiu et al. 2022b [22] Group 1	14 / 15	33.3 / 29.5	Transdiagnostic	DERS		ERQ	OQ-45	WSAS
Neacsiu et al. 2022b [22] Group 2	17 / 15	27.8 / 29.5	Transdiagnostic	DERS		ERQ	OQ-45	WSAS
Osuch et al. 2009 [23]	9 / 9	41.4 / 41.4	PTSD	CAPS	HAM-D			
Palm et al. 2024 [24]	18 / 18	51.4 / 50.6	Addiction	QSU Factor 1				
Ramasawmy et al. 2022 [25]	10 / 10	59.7 / 48.2	Pain	NRS	DASS-21	DASS-21	FIQ	
Ramasawmy et al. 2024 [26]	18 / 19	51.8 / 51.6	Pain	NRS	DASS-21	DASS-21	FIQ	
Tilbor et al. 2024 [27]	8 / 11	51.7 / 46.2	Pain	SF-MPQ	HAM-D			
Witkiewitz et al. 2019 [28]	47 / 37	51.4 / 53.4	Addiction	Drinks per day				SST

ADS: Allegemeine Depressionsskala (English: eneral Depression Scale); ADS-K: Kurzform der Allegemeine Depressionsskala (English: Short form of the General Depression Scale); AQ: Acrophobia Questionnaire; ASI: Anxiety and Sensitivity Index; BAT-1/BAT-2: Behavioral Avoidance Test; BDI: Beck Depression Inventory; BPD: Borderline personality disorder; BSL-23: Borderline Symptom List; CAPS-5: Clinician Administered PTSD Scale; DASS: Depression Anxiety Stress Scales; DERS: Difficulties in Emotion Regulation Scale; EGF/GAF: Global Assessment of Functioning Scale (French/English); ERQ: Emotion Regulation Questionnaire; FIQ: Fibromyalgia Impact Questionnaire; GAD-7: Generalized Anxiety Disorder 7-Item; HAM-A: Hamilton Anxiety Rating Scale; HAM-D: Hamilton Depression Rating Scale; IPF: Inventory of Psychosocial Functioning; MADRS: The Montgomery–Åsberg Depression Rating Scale; MBI: Modified Barthel Index; MDD: Major depressive disorder; MMSE: Mini-Mental State Examination; MoCA: Montreal Cognitive Assessment; NRS: Numerical Rating Scale; OCD: Obsessive-compulsive disorder; OCDS: Obsessive Compulsive Drinking Scale; OQ-45: Outcome Questionnaire; PAS–Observer Rated: Panic and Agoraphobia Scale; PHQ-9: Patient Health Questionnaire; PQSI: Pittsburgh Sleep Quality Index; PRPSA: The Personal Report of Public Speaking Anxiety; PTSD: Post-traumatic stress disorder; QIDS: Quick Inventory of Depressive Symptomology; QSU Factor 1: Questionnaire on Smoking Urges (Factor 1 – intention and desire to smoke); SDS: Sheehan Disability Scale; SER: Questionnaire of Self-Efficacy in Smokers; SF-36-MH: Short-Form 36 – Mental Health; SF-MPQ: Short-form McGill Pain Questionnaire; SST: Stop Signal Task; STAI-A: State-Trait Anxiety Inventory for Adults; SUDS: The Subjective Units of Distress Scale; WCST: Wisconsin Card Sorting Test; WMS: Weschler Memory Scale; WHOQOL-BREFL: World Health Organization Quality of Life Brief; WSAS: The Work and Social Adjustment Scale; Y-BOCS: Yale-Brown Obsessive Compulsive Scale

Table S2: NIBS parameters

**Study**	**NIBS**	**NIBS-therapy timing**	**Target**	**Targeting method**	**Intensity**	**TMS protocol**	**No. NIBS sessions**	**NIBS duration (min)**
Adams et al. 2022 [1]	tDCS	Non-concurrent	Anode over medial PFC	Scalp/EEG	1.5 mA	–	1	20
Aust et al. 2022 [2]	tDCS	Concurrent	Anode over left PFC	Scalp/EEG	2 mA	–	12	30
Cobb et al. 2021 [5]	tDCS	Non-concurrent	Anode over medial PFC	Scalp/EEG	1.7 mA	–	1	20
Eyraud et al. 2024 [10]	tDCS	Concurrent	Anode over left PFC	Scalp/EEG	2 mA	–	10	20
Fayaz Feyzi et al. 2022 [11]	tDCS	Non-concurrent	Anode over left PFC	Scalp/EEG	2 mA	–	16	20
McDonald et al. 2024 [20]	tDCS	Concurrent	Anode over medial PFC	Scalp/EEG	2 mA	–	1	20
Palm et al. 2024 [24]	tDCS	Non-concurrent	Anode over left PFC	Scalp/EEG	2 mA	–	5	20
Ramasawmy et al. 2022 [25]	tDCS	Concurrent	Anode over left M1	Scalp/EEG	2 mA	–	10	20
Ramasawmy et al. 2024 [26]	tDCS	Concurrent	Anode over left M1	Scalp/EEG	2 mA	–	10	20
Witkiewitz et al. 2019 [28]	tDCS	Concurrent	Anode over right PFC	Scalp/EEG	2 mA	–	8	30
Bohmeier et al. 2025 [3]	TMS	Non-concurrent	Left PFC	Scalp/EEG	80% RMT	iTBS	2	3
Carmi et al. 2019 [4]	TMS	Concurrent	Medial PFC	Scalp/EEG	100% RMT	20 Hz	29	18
Cybinski et al. 2024 [6]	TMS	Non-concurrent	Left PFC	sMRI	100% RMT	iTBS	2	3
Deppermann et al. 2017 [7]	TMS	Non-concurrent	Left PFC	Scalp/EEG	80% RMT	iTBS	15	3
Dieler et al. 2014 [8]	TMS	Non-concurrent	Right PFC	Scalp/EEG	80% RMT	iTBS	4	3
Duan et al. 2023 [9]	TMS	Concurrent	Left PFC	Scalp/EEG	80% RMT	10 Hz	20	20
Fitzsimmons et al. 2025 [12] Group 1	TMS	Non-concurrent	Left PFC	fMRI	110% RMT	10 Hz	16	20
Fitzsimmons et al. 2025 [12] Group 2	TMS	Non-concurrent	Left M1	fMRI	110% RMT	10 Hz	16	20
Fryml et al. 2019 [13]	TMS	Concurrent	Left or right PFC	Scalp/EEG	120% RMT	10 Hz	5	30
Herrmann et al. 2017 [14]	TMS	Non-concurrent	Medial PFC	Scalp/EEG	100% RMT	10 Hz	2	20
Hu et al. 2022 [15] Group 1	TMS	Non-concurrent	Right PFC	Scalp/EEG	110% RMT	10 Hz	10	12.5
Hu et al. 2022 [15] Group 2	TMS	Non-concurrent	Left PFC	Scalp/EEG	110% RMT	10 Hz	10	12.5
Isserles et al. 2013 [16]	TMS	Non-concurrent	Medial PFC	Scalp/EEG	120% RMT	20 Hz	12	15.5
Isserles et al. 2021 [17]	TMS	Non-concurrent	Medial PFC	Scalp/EEG	100% RMT	18 Hz	12	30
Kozel et al. 2018 [18]	TMS	Non-concurrent	Right PFC	Scalp/EEG	110% RMT	1 Hz	12	30
Kujovic et al. 2024 [19]	TMS	Non-concurrent	Left PFC	Scalp/EEG	80% RMT	iTBS	20	3
Neacsiu et al. 2022a [21]	TMS	Concurrent	Left PFC	fMRI	120% RMT	10 Hz	1	40
Neacsiu et al. 2022b [22] Group 1	TMS	Concurrent	Left PFC	Scalp/EEG	120% RMT	10 Hz	1	40
Neacsiu et al. 2022b [22] Group 2	TMS	Concurrent	Right PFC	Scalp/EEG	120% RMT	10 Hz	1	40
Osuch et al. 2009 [23]	TMS	Concurrent	Right PFC	Scalp/EEG	100% RMT	1 Hz	20	30
Tilbor et al. 2024 [27]	TMS	Non-concurrent	Medial PFC	Scalp/EEG	100% RMT	20 Hz	20	18

sMRI: structural MRI; fMRI: functional MRI; M1: primary motor cortex; RMT: resting motor threshold; PFC: prefrontal cortex

Table S3: Therapy parameters

**Study**	**Therapy**	**Delivery**	**Concurrent medication allowed**	**Adherence completed**	**No. therapy sessions**	**Therapy duration per session (min)**
Aust et al. 2022 [2]	CBT	Human	Yes	Yes	12	100
Deppermann et al. 2017 [7]	CBT	Human	Yes	No	11	90
Dieler et al. 2014 [8]	CBT	Human	No	No	6	90
Fayaz Feyzi et al. 2022 [11]	CBT	Human	No	No	24	60
Hu et al. 2022 [15]	CBT	Human	No	No	8	60
Kozel et al. 2018 [18]	CBT	Human	Yes	No	12	60
Kujovic et al. 2024 [19]	CBT	Human	Yes	Yes	8 wks total (1–2 sessions/wk)	Variable
Neacsiu et al. 2022a [21]	CBT	Hybrid	Yes	No	1	36
Neacsiu et al. 2022b [22]	CBT	Hybrid	Yes	No	1	36
Palm et al. 2024 [24]	CBT	Human	No	No	5	5
Adams et al. 2022 [1]	Exposure	Human	Yes	No	2	50
Bohmeier et al. 2025 [3]	Exposure	Hybrid	No	No	2	27.3
Carmi et al. 2019 [4]	Exposure	Human	Yes	No	29	3–5
Cobb et al. 2021 [5]	Exposure	Human	Yes	Yes	1	30
Cybinski et al. 2024 [6]	Exposure	Hybrid	No	No	2	28.9
Eyraud et al. 2024 [10]	Exposure	Computerized	Yes	No	10	20
Fitzsimmons et al. 2025 [12]	Exposure	Human	Yes	No	20	60
Fryml et al. 2019 [13]	Exposure	Human	Yes	Unclear	5	40
Herrmann et al. 2017 [14]	Exposure	Computerized	No	Unclear	2	50
Isserles et al. 2013 [16]	Exposure	Computerized	Yes	No	12	4
Isserles et al. 2021 [17]	Exposure	Computerized	Yes	No	12	1.5
McDonald et al. 2024 [20]	Exposure	Computerized	Yes	No	1	18
Osuch et al. 2009 [23]	Exposure	Hybrid	Yes	No	20	5
Duan et al. 2023 [9]	Mindfulness	Human	Yes	No	6	60
Ramasawmy et al. 2022 [25]	Mindfulness	Hybrid	Yes	No	10	30
Ramasawmy et al. 2024 [26]	Mindfulness	Hybrid	Yes	No	10	20
Tilbor et al. 2024 [27]	Mindfulness	Hybrid	Yes	No	20	20
Witkiewitz et al. 2019 [28]	Mindfulness	Human	No	No	8	120

CBT: cognitive behavioral therapy

References

[1] T. G. Adams, J. M. Cisler, B. Kelmendi, J. R. George, S. A. Kichuk, C. L. Averill, A. Anticevic, C. G. Abdallah, and C. Pittenger. Transcranial direct current stimulation targeting the medial prefrontal cortex modulates functional connectivity and enhances safety learning in obsessive-compulsive disorder: Results from two pilot studies. *Depress Anxiety*, 39(1):37–48, 2022. doi: 10.1002/da.23212.

[2] S. Aust, E.-L. Brakemeier, J. Spies, A. L. Herrera-Melendez, T. Kaiser, A. Fallgatter, C. Plewnia, S. V. Mayer, E. Dechantsreiter, G. Burkhardt, M. Strauß, N. Mauche, C. Normann, L. Frase, M. Deuschle, A. Böhringer, F. Padberg, and M. Bajbouj. Efficacy of augmentation of cognitive behavioral therapy with transcranial direct current stimulation for depression: A randomized clinical trial. *JAMA Psychiatry*, 79(6):528–537, 2022. doi: 10.1001/jamapsychiatry.2022.0696.

[3] B. Bohmeier, L. M. Cybinski, D. Gromer, D. Bellinger, J. Deckert, A. Erhardt-Lehmann, L. Deserno, A. Mühlberger, P. Pauli, T. Polak, and M. J. Herrmann. Intermittent theta burst stimulation of the left dorsolateral prefrontal cortex has no additional effect on the efficacy of virtual reality exposure therapy for acrophobia. a randomized double-blind placebo-controlled study. *Behav Brain Res*, 476:115232, 2025. doi: 10.1016/j.bbr.2024.115232.

[4] L. Carmi, A. Tendler, A. Bystritsky, E. Hollander, D. M. Blumberger, J. Daskalakis, H. Ward, K. Lapidus, W. Goodman, L. Casuto, D. Feifel, N. Barnea-Ygael, Y. Roth, A. Zangen, and J. Zohar. Efficacy and safety of deep transcranial magnetic stimulation for obsessive-compulsive disorder: A prospective multicenter randomized double-blind placebo-controlled trial. *Am J Psychiatry*, 176(11):931–938, 2019. doi: 10.1176/appi.ajp.2019.18101180.

[5] A. R. Cobb, P. O'Connor, E. Zaizar, K. Caulfield, F. Gonzalez-Lima, and M. J. Telch. tDCS-augmented in vivo exposure therapy for specific fears: A randomized clinical trial. *J Anxiety Disord*, 78:102344, 2021. doi: 10.1016/j.janxdis.2020.102344.

[6] L. M. Cybinski, B. Bohmeier, K. Rolle, D. Gromer, T. Raij, F. Gundelach, A. Erhardt-Lehmann, A. Mühlberger, J. Deckert, T. Polak, P. Pauli, and M. J. Herrmann. Intermittent theta burst stimulation over the left prefrontal cortex: no additional effect for virtual reality exposure therapy in acrophobia-a randomized trial. *Sci Rep*, 14(1):29450, 2024. doi: 10.1038/s41598-024-80832-1.

[7] S. Deppermann, N. Vennewald, J. Diemer, S. Sickinger, F. B. Haeussinger, T. Dresler, S. Notzon, I. Laeger, V. Arolt, A. C. Ehlis, A. J. Fallgatter, and P. Zwanzger. Neurobiological and clinical effects of fNIRS-controlled rTMS in patients with panic disorder/agoraphobia during cognitive-behavioural therapy. *Neuroimage Clin*, 16:668–677, 2017. doi: 10.1016/j.nicl.2017.09.013.

[8] A. C. Dieler, T. Dresler, K. Joachim, J. Deckert, M. J. Hermann, and A. J. Fallgatter. Can intermittent theta burst stimulation as add-on to psychotherapy improve nicotine abstinence? Results from a pilot study. *Eur Addict Res*, 20 (5):248–253, 2014. doi: 10.1159/000357941.

[9] H. Duan, X. Yan, S. Meng, L. Qiu, J. Zhang, C. Yang, and S. Liu. Effectiveness evaluation of repetitive transcranial magnetic stimulation therapy combined with mindfulness-based stress reduction for people with post-stroke depression: A randomized controlled trial. *Int J Environ Res Public Health*, 20(2):930, 2023. doi: 10.3390/ijerph20020930.

[10] N. Eyraud, P. Poupin, M. Legrand, A. Caille, A. Sauvaget, S. Bulteau, B. Gohier, G. Harika-Germaneau, D. Drapier, N. Jaafari, O. Bodic, B. Brizard, V. Gissot, C. Belzung, J. B. Courtine, and W. El-Hage. Combining trauma script exposure with tDCS to alleviate symptoms of posttraumatic stress disorder: A two-arm randomized sham-controlled multicenter trial. *Brain Stimul*, 17(3):591–593, 2024. doi: 10.1016/j.brs.2024.04.018.

[11] Y. Fayaz Feyzi, N. Vahed, N. Sadeghamal Nikraftar, and R. Arezoomandan. Synergistic effect of combined transcranial direct current stimulation and Matrix Model on the reduction of methamphetamine craving and improvement of cognitive functioning: A randomized sham-controlled study. *Am J Drug Alcohol Abuse*, 48(3):311–320, 2022. doi: 10.1080/00952990.2021.2015771.

[12] S. M. D. D. Fitzsimmons, T. S. Postma, A. D. van Campen, C. Vriend, N. M. Batelaan, P. van Oppen, A. W. Hoogendoorn, Y. D. van der Werf, and O. A. van den Heuvel. Transcranial magnetic stimulation-induced plasticity improving cognitive control in obsessive-compulsive disorder, Part I: clinical and neuroimaging outcomes from a randomized trial. *Biol Psychiatry*, 97(7):678–687, 2025. doi: 10.1016/j.biopsych.2024.06.029.

[13] L. D. Fryml, C. G. Pelic, R. Acierno, P. Tuerk, M. Yoder, J. J. Borckardt, N. Juneja, M. Schmidt, K. L. Beaver, and M. S. George. Exposure therapy and simultaneous repetitive transcranial magnetic stimulation: A controlled pilot trial for the treatment of posttraumatic stress disorder. *J ECT*, 35(1):53–60, 2019. doi: 10.1097/YCT.0000000000000505.

[14] M. J. Herrmann, A. Katzorke, Y. Busch, D. Gromer, T. Polak, P. Pauli, and J. Deckert. Medial prefrontal cortex stimulation accelerates therapy response of exposure therapy in acrophobia. *Brain Stimul*, 10(2):291–297, 2017. doi: 10.1016/j.brs.2016.11.007.

[15] X. Hu, T. Zhang, H. Ma, X. Zhou, H. Wang, X. Wang, C. Cheng, Y. Li, R. Duan, B. Zhang, H. Wang, J. Lu, C. Kang, N. Zhao, Y. Zhang, L. Tian, J. Liu, J. Shi, Z. Wang, X. Zhou, S. Zhu, Q. Liu, X. Li, H. Wang, M. Nie, M. Yang, J. Yang, Y. Chi, X. Zhu, J. Hu, Y. Jia, Y. Peng, and L. Liu. Repetitive transcranial magnetic stimulation combined with cognitive behavioral therapy treatment in alcohol-dependent patients: A randomized, double-blind sham-controlled multicenter clinical trial. *Front Psychiatry*, 13:935491, 2022. doi: 10.3389/fpsyt.2022.935491.

[16] M. Isserles, A. Y. Shalev, Y. Roth, T. Peri, I. Kutz, E. Zlotnick, and A. Zangen. Effectiveness of deep transcranial magnetic stimulation combined with a brief exposure procedure in post-traumatic stress disorder–a pilot study. *Brain Stimul*, 6(3):377–383, 2013. doi: 10.1016/j.brs.2012.07.008.

[17] M. Isserles, A. Tendler, Y. Roth, A. Bystritsky, D. M. Blumberger, H. Ward, D. Feifel, L. Viner, W. Duffy, J. Zohar, C. J. Keller, M. T. Bhati, A. Etkin, M. S. George, I. Filipcic, K. Lapidus, L. Casuto, S. Vaishnavi, A. Stein, L. Deutsch, F. Deutsch, O. Morales, Z. J. Daskalakis, A. Zangen, and K. J. Ressler. Deep transcranial magnetic stimulation combined with brief exposure for posttraumatic stress disorder: A prospective multisite randomized trial. *Biol Psychiatry*, 90 (10):721–728, 2021. doi: 10.1016/j.biopsych.2021.04.019.

[18] F. A. Kozel, M. A. Motes, N. Didehbani, B. DeLaRosa, C. Bass, C. D. Schraufnagel, P. Jones, C. R. Morgan, J. S. Spence, M. A. Kraut, and J. Hart, Jr. Repetitive TMS to augment cognitive processing therapy in combat veterans of recent conflicts with PTSD: A randomized clinical trial. *J Affect Disord*, 229:506–514, 2018. doi: 10.1016/j.jad.2017.12.046.

[19] M. Kujovic, C. Bahr, M. Riesbeck, D. Benz, L. Wingerter, M. Deiß, Z. Margittai, D. Reinermann, C. Plewnia, and E. Meisenzahl. Effects of intermittent theta burst stimulation add-on to dialectical behavioral therapy in borderline personality disorder: Results of a randomized, sham-controlled pilot trial. *Eur Arch Psychiatry Clin Neurosci*, 2024. doi: 10.1007/s00406-024-01901-0. Online ahead of print.

[20] M. A. McDonald, S. J. Meckes, J. Shires, M. E. Berryhill, and C. L. Lancaster. Augmenting virtual reality exposure therapy for social and intergroup anxiety with transcranial direct current stimulation. *J ECT*, 40(1):51–60, 2024. doi: 10.1097/YCT.0000000000000967.

[21] A. D. Neacsiu, L. Beynel, J. L. Graner, S. T. Szabo, L. G. Appelbaum, M. J. Smoski, and K. S. LaBar. Enhancing

cognitive restructuring with concurrent fMRI-guided neurostimulation for emotional dysregulation - A randomized controlled trial. *J Affect Disord*, 301:378–389, 2022. doi: 10.1016/j.jad.2022.01.053.

[22] A. D. Neacsiu, L. Beynel, J. P. Powers, S. T. Szabo, L. G. Appelbaum, S. H. Lisanby, and K. S. LaBar. Enhancing cognitive restructuring with concurrent repetitive transcranial magnetic stimulation: A transdiagnostic randomized controlled trial. *Psychother Psychosom*, 91(2):94–106, 2022. doi: 10.1159/000518957.

[23] E. A. Osuch, B. E. Benson, D. A. Luckenbaugh, M. Geraci, R. M. Post, and U. McCann. Repetitive TMS combined with exposure therapy for PTSD: A preliminary study. *J Anxiety Disord*, 23(1):54–59, 2009. doi: 10.1016/j.janxdis.2008.03.015.

[24] U. Palm, M. Obergfell, A. Rabenstein, J. Björklund, G. Koller, F. Padberg, and T. Rüther. Transcranial direct current stimulation combined with a brief intervention for smoking cessation: A randomized double-blind clinical trial. *Eur Arch Psychiatry Clin Neurosci*, 274(4):1001–1011, 2024. doi: 10.1007/s00406-023-01705-8.

[25] P. Ramasawmy, S. Khalid, F. Petzke, and A. Antal. Pain reduction in fibromyalgia syndrome through pairing transcranial direct current stimulation and mindfulness meditation: A randomized, double-blinded, sham-controlled pilot clinical trial. *Front Med*, 9:908133, 2022. doi: 10.3389/fmed.2022.908133.

[26] P. Ramasawmy, O. L. Gamboa Arana, T. T. Mai, L. C. Heim, S. E. Schumann, E. Fechner, Y. Jiang, O. Moschner, I. Chakalov, M. Bähr, F. Petzke, and A. Antal. No add-on therapeutic benefit of at-home anodal tDCS of the primary motor cortex to mindfulness meditation in patients with fibromyalgia. *Clin Neurophysiol*, 164:168–179, 2024. doi: 10.1016/j.clinph.2024.05.018.

[27] E. Tilbor, A. Hadar, V. Portnoy, O. Ganor, Y. Braw, H. Amital, J. Ablin, C. Dror, Y. Bloch, and U. Nitzan. TMS in combination with a pain directed intervention for the treatment of fibromyalgia - A randomized, double-blind, sham-controlled trial. *J Psychiatr Res*, 170:167–173, 2024. doi: 10.1016/j.jpsychires.2023.12.016.

[28] K. Witkiewitz, E. R. Stein, V. R. Votaw, A. D. Wilson, C. R. Roos, S. J. Gallegos, V. P. Clark, and E. D. Claus. Mindfulness-based relapse prevention and transcranial direct current stimulation to reduce heavy drinking: A double-blind sham-controlled randomized trial. *Alcohol Clin Exp Res*, 43(6):1296–1307, 2019. doi: 10.1111/acer.14053.
